# Small Molecule Inhibitors of Plasminogen Activator Inhibitor-1 Elicit Anti-Tumorigenic and Anti-Angiogenic Activity

**DOI:** 10.1371/journal.pone.0133786

**Published:** 2015-07-24

**Authors:** Veronica R. Placencio, Atsuhiko Ichimura, Toshio Miyata, Yves A. DeClerck

**Affiliations:** 1 Division of Hematology, Oncology and Blood and Bone Marrow Transplantation, Department of Pediatrics, University of Southern California, Los Angeles, California, United States of America; 2 The Saban Research Institute of Children’s Hospital, Los Angeles, California, 90027, United States of America; 3 Tohoku University Graduate School of Medicine, Miyagi, Japan; 4 Department of Biochemistry and Molecular Biology, University of Southern California, Los Angeles, California, United States of America; Institute of Biochemistry and Biotechnology, TAIWAN

## Abstract

Numerous studies have shown a paradoxical positive correlation between elevated levels of plasminogen activator inhibitior-1 (PAI-1) in tumors and blood of cancer patients with poor clinical outcome, suggesting that PAI-1 could be a therapeutic target. Here we tested two orally bioavailable small molecule inhibitors of PAI-1 (TM5275 and TM5441) for their efficacy in pre-clinical models of cancer. We demonstrated that these inhibitors decreased cell viability in several human cancer cell lines with an IC_50_ in the 9.7 to 60.3 μM range and induced intrinsic apoptosis at concentrations of 50 μM. *In vivo*, oral administration of TM5441 (20 mg/kg daily) to HT1080 and HCT116 xenotransplanted mice increased tumor cell apoptosis and had a significant disruptive effect on the tumor vasculature that was associated with a decrease in tumor growth and an increase in survival that, however, were not statistically significant. Pharmacokinetics studies indicated an average peak plasma concentration of 11.4 μM one hour after oral administration and undetectable levels 23 hours after administration. The effect on tumor vasculature *in vivo* was further examined in endothelial cells (EC) *in vitro* and this analysis indicated that both TM5275 and TM5441 inhibited EC branching in a 3D Matrigel assay at concentrations where they had little effect on EC apoptosis. These studies bring novel insight on the activity of PAI-1 inhibitors and provide important information for the future design of inhibitors targeting PAI-1 as therapeutic agents in cancer.

## Introduction

Plasminogen activator inhibitor-1 (PAI-1) is a serine protease inhibitor that plays an important role in many physiological and pathological conditions, including wound healing, obesity, metabolic syndrome, cardiovascular disease and cancer [1]. PAI-1 has a dual function. It inhibits urokinase plasminogen activator (uPA) and tissue plasminogen activator (tPA) to prevent plasminogen cleavage into active plasmin and blocks fibrinolysis [1, 2]. Also, it binds to the somatomedin B domain of vitronectin to prevent integrin-mediated binding to the tripeptide Arg-Gly-Asp (RGD) domain of vitronectin [3]. In cancer patients, many studies have reported a paradoxically positive correlation between elevated levels of PAI-1 in tumors and blood with poor clinical outcome [4, 5]. This paradoxical effect of PAI-1 has since been explained by its pro-angiogenic activity and its protective effect on cell apoptosis. Studies using physiological levels of PAI-1 revealed that it stimulates endothelial cell (EC) migration and proliferation through its anti-protease activity and its ability to bind to vitronectin causing EC to migrate from the vitronectin-rich perivascular space towards fibronectin-rich tumor stroma [6, 7]. We have also shown that PAI-1 protects EC from Fas ligand (Fas-L)-dependent extrinsic apoptosis [8]. *In vivo*, several animal studies in PAI-1 deficient mice have confirmed that a lack of PAI-1 in host cells and in tumor cells inhibits angiogenesis and enhances apoptosis [9, 10]. Together these data suggest that PAI-1 could be a target for therapeutic intervention. However, this possibility has not been well explored so far.

Several small molecule inhibitors of PAI-1 were developed over the last 20 years and tested primarily for their pre-clinical efficacy in promoting vascular re-permeabilization in models of acute thrombosis and tissue fibrosis. A first inhibitor, PAI-039 (Tiplaxtinin) was shown to accelerate thrombus re-permeabilization in rats and dogs after acute carotid injury [11]. In xenotransplanted T24 bladder tumors, it inhibits angiogenesis and induces apoptosis leading to a significant reduction in tumor growth [12]. However this inhibitor was not developed further clinically, in part because its lack of activity against vitronectin bound PAI-1, which is the stable form of PAI-1 [13].

More recently carboxylic acid-derived small molecule inhibitors with activity against PAI-1 have been synthesized, including first generation dimeric 2-acylamino-3-thiophenecarboxylic acid derivatives (TM5001 and TM5007) [14]. These compounds have shown efficacy in rodent models of thrombosis and lung fibrosis, however they had poor solubility and poor absorption [14]. A second generation PAI-1 inhibitor, TM5275, with increased solubility and better oral absorption was developed [15]. When tested in pre-clinical models of vascular thrombosis in rat and non-human primates, this inhibitor was shown to have an anti-thrombotic effect [15]. It also had anti-fibrotic activity in murine models of transforming growth factor β-induced lung fibrosis [16]. A third molecule, TM5441, with better pharmacokinetics and volume of distribution was more recently developed and further described [17, 18]. When administered to mice treated with nitric oxide synthase inhibitors L-Nitroarginine-Methyl-Ester (L-NAME), it inhibited hypertension, cardiac hypertrophy and vascular fibrosis [17]. The effect of these carboxylic acid-derived inhibitors in cancer has not been fully explored. However, a recent report showing that TM5275 induces apoptosis *in vitro* in ovarian cancer cells suggests that these inhibitors may also have an anti-cancer activity [19]. Here we tested the *in vitro* activity of TM5275 and TM5441 against a large variety of human tumor cell lines and the pre-clinical efficacy of TM5441 *in vivo* in HT1080 and HCT116 tumor-bearing mice. Our data demonstrate the *in vitro* apoptotic effect of these inhibitors against several tumor cell lines but point to their present limited activity when used alone *in vivo*. Our studies, the first on the use of these inhibitors *in vivo*, bring important insight into their potential, yet current limitations, of these agents as therapeutic and anti-angiogenic drugs in cancer while providing information for the design of future PAI-1 inhibitors.

## Materials and Methods

### Cell culture

HT1080 (fibrosarcoma), HCT116 (colorectal carcinoma), Daoy (desmoplastic cerebellar medulloblastoma), MDA-MB-231 (breast adenocarcinoma), and Jurkat (T lymphocytes from acute lymphocytic leukemia) human cancer cell lines were purchased from ATCC. They were grown in DMEM medium supplemented with 10% fetal bovine serum. HUVEC (human umbilical vein endothelial cells) were purchased from AllCells and maintained as recommended. HT1080 and HCT116 cells were cultured in DMEM medium containing 5% NuSerum (Becton Dickinson) with a final concentration of 1.25% newborn calf serum. These cells were authenticated every 6 months by short tandem repeat profiling analysis.

### Small molecule inhibitors

TM5275 and TM5441 inhibitors were obtained as lyophilized powder in amber glass vials and kept in the dark at room temperature [15]. TM5275 was synthesized as previously reported [20] and synthesis information is available for TM5441 [patent number US 8415479 (Compound 58, example 58) or WO2009-123241 (Example 58)]. They were dissolved with dimethyl sulfoxide (DMSO) at a stock concentration of 50 mM, stored at -20°C, and diluted in media for *in vitro* experiments. For *in vivo* experiments, TM5441 (20, 50 or 100 mg/kg) was dissolved in DMSO and incorporated into individual servings of peanut butter and honey. Controls were given equal amounts of vehicle (equal volumes of DMSO mixed in peanut butter and honey). Each mouse was then administered the inhibitor or vehicle mixture until it had eaten the entire dose.

### Cell viability assay

Cell lines were plated in quadruplicate wells overnight in 96-well plates at a density of 6,000 cells per well and treated the next day. The cells were incubated for 48 hours at 37°C. The CellTiter-Glo luminescent cell viability assay (Promega) was used according to the manufacturer’s recommendations. Viability (expressed as a % of control to DMSO treated cells) was plotted on a logarithmic scale and the half maximal inhibitor concentration (IC_50_) was calculated from the best fit line.

### Flow cytometry

Cells were plated in triplicate in 6-well plates at a density of 120,000 cells per well and treated with 50 μM TM5275 or TM5441 the next day for eight hours (BromodeoxyUridine (BrdU) incorporation) or 24 and 48 hours (mitochondrial depolarization). For Annexin V, cells were treated with the indicated doses for 48 hours. For BrdU incorporation, cells were pulsed with 10 μM BrdU for 20 minutes before being harvested using the fluorescein isothiocyanate (FITC) BrdU Flow kit (BD) according to the manufacturer’s recommendations. Mitochondrial depolarization was assessed using the MitoProbe 5,5′,6,6′-tetrachloro-1,1′,3,3′-tetraethylbenzimidazolylcarbocyanine iodide (JC-1) assay kit (Life Technologies) according to the manufacturer’s recommendations. Apoptotic cells (early apoptotic Annexin V^+^/PI^-^ cells and late apoptotic Annexin V^+^/PI^+^ cells) were evaluated using the Annexin V FITC apoptosis detection kit I (BD) according to the manufacturer’s recommendations. The cells were analyzed by flow cytometry in a BD LSR II system (BD) with DiVA software (version 6.0, BD).

### Caspase 3/7 activity assay

Cells were plated as described for cell viability and treated with increasing concentrations of TM5275 or TM5441 for 48 hours. The ApoLive-Glo kit (Promega) was used to measure cell viability with a fluorescent dye followed by the measurement of caspase 3/7 activity with luminescence activity according to the manufacturer’s recommendations at room temperature. Caspase 3/7 activity was normalized to cell viability and plotted as fold change compared to DMSO control cells.

### Cell-associated plasmin activity assay

Human plasmin activity in whole cell lysates was determined using a chromogenic plasmin substrate D-VLK-pNA (Molecular Innovations) as previously described. Cells were cultured in 5% acidified NuSerum (to suppress non-specific protease inhibition by α2-macroglobulin) and cell lysates were incubated with the substrate overnight at 37°C [10].

### Immunoblot analysis

Samples (30 μg) were electrophoresed in sodium dodecyl sulfate (SDS)-polyacrylamide gels, which were transferred to nitrocellulose membranes and probed for the following proteins: caspase 8 (mouse monoclonal antibody (mAb), 1:1,000 dilution, Cell Signaling Technology), caspase 9 (rabbit polyclonal 1:1000 dilution, Cell Signaling Technology), caspase 3 (mouse mAb 1:1,000 dilution, Cell Signaling Technology), poly ADP ribose polymerase (PARP) (rabbit polyclonal 1:1,000 dilution, Cell Signaling Technology), and Tubulin (mouse mAb 1:1,000 dilution, Sigma Aldrich). IRDye 800CW or IRDye 680RD secondary antibodies (1:10,000 dilutions) were used to visualize protein on the Odyssey Imaging system (LI-COR). Protein band pixel density was quantitated (Image Studio version 3.1 software) and the ratio of the cleaved protein band compared to the tubulin loading control band was used to compare across conditions.

### Endothelial cell branching assay

Ninety-six-well plates were coated with 50 μL Matrigel (Corning) and 20,000 HUVEC cells were plated in triplicate wells for each condition. After 24 hours, the total number of branched “Y” structures were counted per well. Light microscopy images were taken on a Leica DM IL LED microscope.

### Plasma TM concentration analysis

Retro-orbital blood collection was performed using heparinized tubes one hour after inhibitor administration for peak plasma concentrations and one hour prior to next dose for trough plasma concentrations. End point blood was also drawn one hour after the last inhibitor dose prior to euthanasia. Twenty-five μL of plasma was used for reverse-phase high performance liquid chromatography analysis [15].

### Xenograft mouse models

HT1080 cells were transduced with a firefly luciferase lentiviral vector (Genecopoeia) and selected against 100 μg/ mL geneticin for two weeks. Cells were then maintained with geneticin. Five-week-old nu/nu female mice (The Jackson Laboratory) were injected with 5x10^6^ HT1080-luciferase cells subcutaneously into the right flank as described previously [10]. Palpable tumors and mouse weight were measured every 2–3 days. Mice were sacrificed when tumor volume reached a maximum of 1,500 mm^3^ calculated with the modified ellipsoid formula: tumor volume (mm^3^) = (width in mm)^2^ X (length in mm) X π/6 [10]. For bioluminescence imaging, mice were injected intravenously (i.v.) with D-luciferin (0.3 mL) at a dose of 5 mg/kg and luciferase activity was determined after 15 minutes with a two-second exposure time using the Xenogen IVIS system [21]. Bioluminescence emitted from the HT1080 tumor cells (total flux) was measured and plotted as relative luciferase units. For bleeding time testing, the dorsal tail vein was cut using an automated Surgicutt Newborn device (International Technidyne Corporation) 0.5 mm deep and 2.5 mm long. Filter paper was carefully blotted against the bleeding wound every 15 seconds until the incision stopped bleeding. Bleeding time was performed on the last day one hour after administration of the last dose of TM5441 and before the mice were sacrificed (at day 16–30). Measurements were recorded to the nearest 15 seconds.

### Histological analysis of tumors

H&E staining was performed on 4% paraformaldehyde fixed and paraffin embedded tumor sections (5 μm thick). Terminal deoxynucleotidyl transferase dUTP nick end labeling (TUNEL) staining was executed according to the manufacturer’s recommendations using the ApopTag Peroxidase *In Situ* Apoptosis Detection kit (Millipore). Bright field images were taken on a Zeiss wide-field Axioplan microscope equipped with a 40×/1.3 oil Plan-NEOFLUAR lens (Carl Zeiss Microimaging) and SPOT Insight QE color camera (Diagnostic Instruments). TUNEL optical density was quantitated by taking the ratio of TUNEL positive staining to the total tissue area. CD31 (1:100, Abcam) immunofluorescence staining was performed with antigen retrieval using 20 mg/kg proteinase K. An anti-rat Alexa Fluor 647 secondary antibody was used (1:300, Life Technologies). Images were taken with an Axiovert 200M microscope equipped with a 20x/0.8 Plan-APOCHROMAT lens (Carl Zeiss Microimaging) and ORCA-AG camera (Hamamatsu Photonics) using Micro-Manager software[22]. CD31 blood vessel density was quantitated by taking the ratio of CD31-red positive staining to the total DAPI-blue positive staining.

### Statistics

A zero plateau sigmoidal model was utilized to fit the cell viability data. The half maximal inhibitory concentration, or IC_50_, was extracted from each non-linear model. The difference in mouse survival time between the tumor injection and death was evaluated using linear regression. Tumor volume and flux measurements from the *in vivo* experiments were log_10_ transformed to minimize outlier effects. The area under the curve (AUC) was calculated for statistical evaluation using the transformed tumor growth values up to the time point with the most observed tumor measurements. Differences in AUC of the flux among the treatment groups were evaluated with censored normal regression, and the two-sample Wilcoxon rank sum test was used to assess differences in AUC of the tumor volume. The *in vivo* results were verified with permutation tests (10,000 replicates). All other comparisons were evaluated with analysis of variance (ANOVA) and when appropriate the results were adjusted for multiple comparisons. The cell viability and *in vivo* data were completed in R (version 3.0.3) [23] and Stata (version 11.2) [24] software, respectively. All other analyses were performed in GraphPad Prism (version 6.05). Unless otherwise stated, all tests refer to two-sided tests, with α = 0.05.

## Results

### TM5275 and TM5441 decreased survival of cancer cells

**Fig 1:**
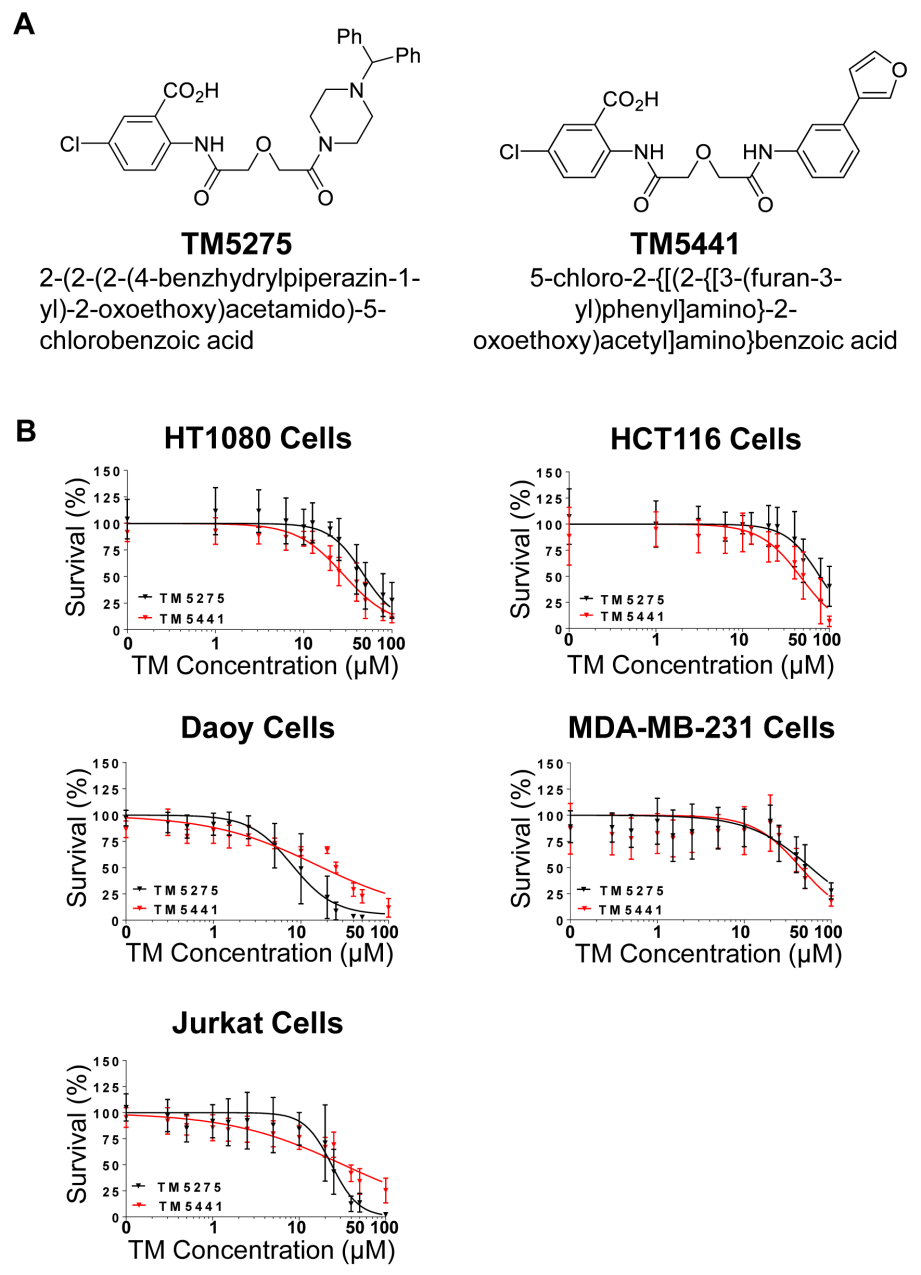
Decreased cell viability in cancer cells treated with TM5275 and TM5441. **A.** The structures and chemical names of TM5275 and TM5441 are shown. **B.** The indicated cell lines were treated with increasing concentrations of TM5275 (black) or TM5441 (red). Viability was measured by relative luminescence units for increasing concentrations of TM5275 and TM5441 and were graphed as % survival (± SD) in comparison to DMSO control treated cells. Data was plotted and a best fit line was drawn, n = 3.

We first screened 17 human cell lines (14 malignant and 3 non-malignant) for their *in vitro* sensitivity to TM5275 and TM5441 with a cell viability assay. This analysis indicated a significant dose-dependent decrease in cell viability in the presence of TM5275 and TM5441 in several cell lines (HT1080, HCT116, Daoy, MDA-MB-231 and Jurkat) with an IC_50_ ranging between 9.7 and 60.3 μM (Fig 1 and S1 Table). However many other malignant and non-malignant cell lines were resistant to TM5275 showing an IC_50_ above 50 μM (S1 Fig). We selected the HT1080 and HCT116 cell lines for further studies because they showed the lowest TM5441 IC_50_ and because previous work in the laboratory had demonstrated a pro-tumorigenic role of PAI-1 in these cell lines [10].

### TM5275 and TM5441 decreased proliferation of HT1080 and HCT116 cells

**Fig 2:**
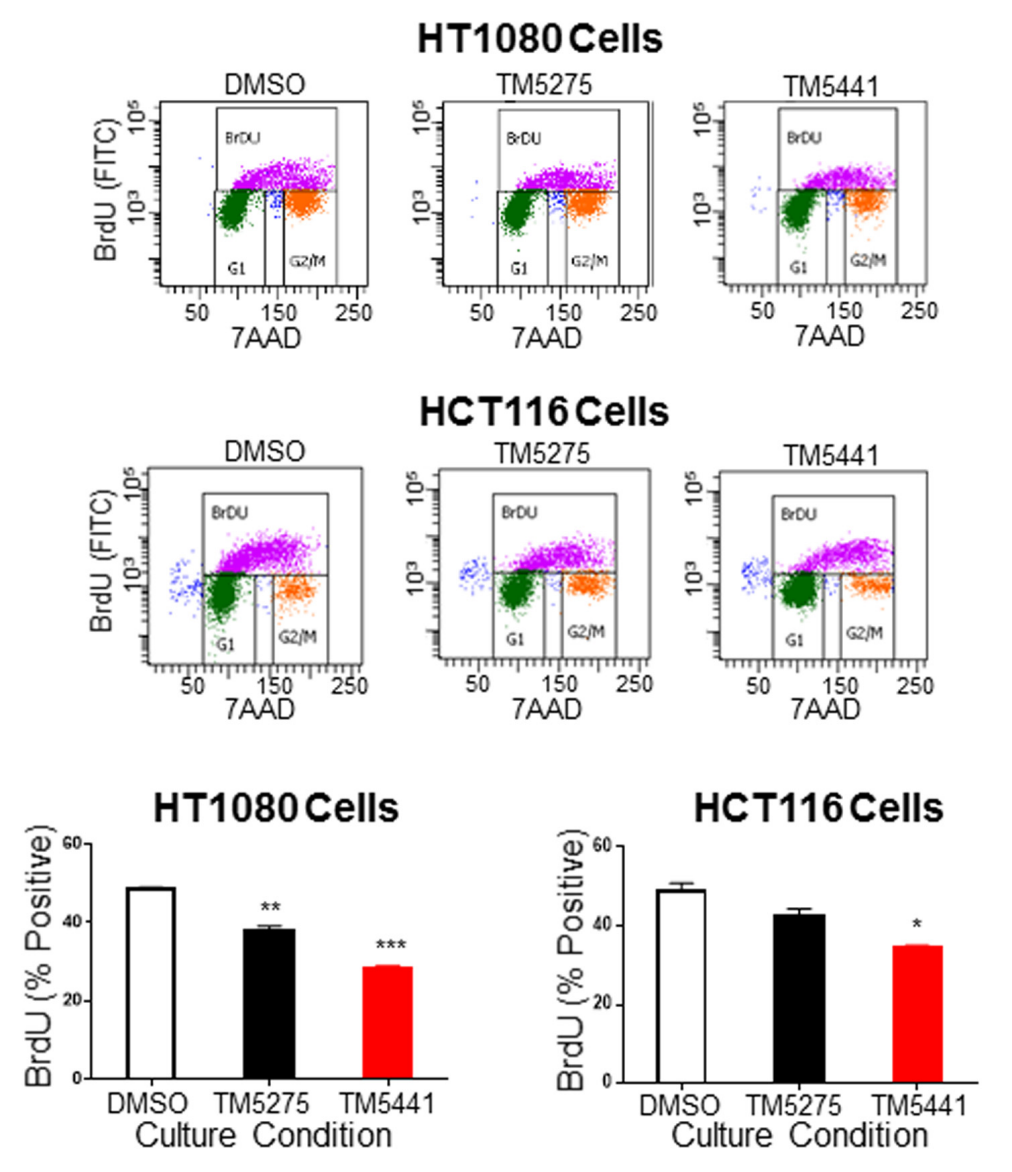
Decreased proliferation in cancer cells treated with TM5275 and TM5441. Representative fluorescence-activated cell sorting (FACS) plots are shown in the upper panels with BrdU positive cells (purple) shown within the upper gate. The mean percentage (± SD) of HT1080 and HCT116 BrdU positive cells after treatment with DMSO (white bar), TM5275 (black bar) or TM5441 (red bar) was graphed, n = 3. * indicates *P* values compared to DMSO controls < 0.05, ** indicates *P* < 0.01, and *** indicates *P* < 0.001.

To examine the effect of TM inhibitors on cell proliferation, we used BrdU incorporation to test cell cycle activity. This analysis (Fig 2) revealed a significant decrease in the percentage of BrdU positive cells with both cell lines treated with TM5275 and TM5441 (from 48.5% and 48.7% in DMSO-treated cells to 38.1% and 42.5% in TM5275-treated HT1080 and HCT116 cells and 28.3% and 34.6% for TM5441-treated cells, respectively). These results indicated that TM5275 and TM5441 decreased tumor cell viability in part through diminished proliferation.

### TM5275 and TM5441 increased apoptosis in HT1080 and HCT116 cells

**Fig 3:**
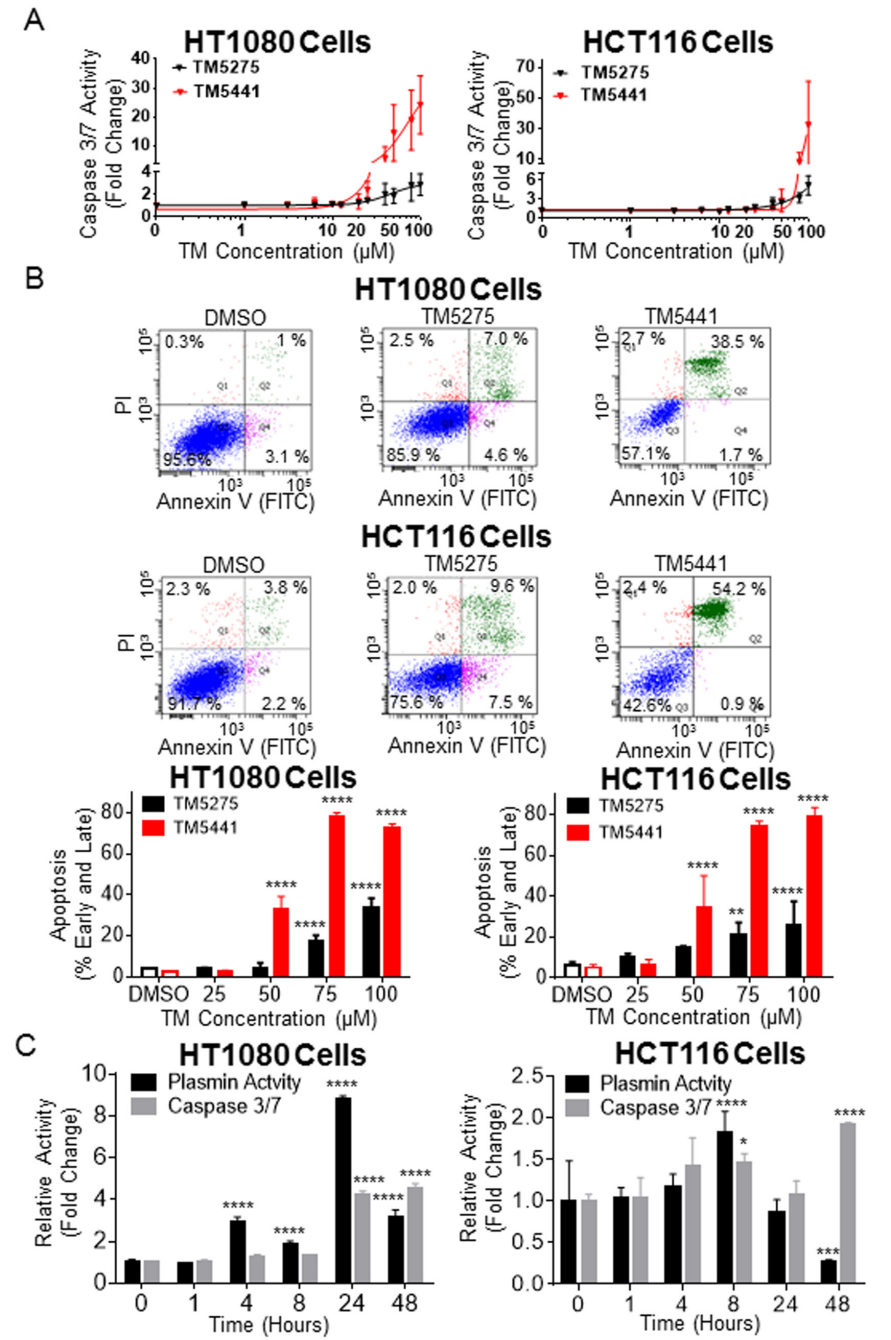
Increased apoptosis in cancer cells treated with TM5275 and TM5441. **A**. Caspase 3/7 activity of HT1080 and HCT116 cells treated with increasing concentrations of TM5275 (black) or TM5441 (red) after 48 hours was plotted as the average fold change (± SD) compared to DMSO-treated cells, n = 3. **B**. Representative FACS plots of HT1080 and HCT116 cells treated with DMSO, TM5275 (50 μM) and TM5441 (50 μM) are shown in the upper panels with annexin V-FITC and propidium iodide staining. The average percentage of cells in early (Annexin V positive, PI negative) and late (Annexin V and PI positive) apoptosis (± SD) was graphed for the indicated concentrations of TM5275 (black bars) or TM5441 (red bars) in each cell line, n = 3. **C**. Average plasmin activity (black bars, ± SD) and average caspase 3/7 activity (grey bars, ± SD) was plotted as a fold change for 50 μM TM5275-treated cells compared to control DMSO-treated cells for each time point, n = 2. * indicates *P* values compared to DMSO controls < 0.05, *** indicates *P* < 0.001, and **** indicates *P* < 0.0001.

Because previous studies indicated that PAI-1 protects tumor cells from apoptosis [10, 12, 25, 26] we examined the effect of TM5275 and TM5441 on apoptosis in HT1080 and HCT116 cells. Using a caspase 3/7 activity assay, we demonstrated a dose-dependent increase in caspase 3/7 activity for both HT1080 and HCT116 cells exposed to these inhibitors (Fig 3A). The effect on caspase 3/7 activity was statistically much stronger with TM5441 (37-fold and 32-fold, respectively, at 100 μM) than with TM5275 (3-fold and 5-fold; *P* value = 0.0005 and 0.003, respectively). The effect of the inhibitors on apoptosis was confirmed by analysis of Annexin V and propidium iodide (PI) staining by flow cytometry (Fig 3B). The data indicated a statistically significant dose-dependent increase in early and late apoptosis in HT1080 and HCT116 cells treated with TM5275 or TM5441 inhibitors when compared with DMSO-treated cells. As a readout for PAI-1 activity against uPA, we measured plasmin activity over time. This analysis revealed an increase in cell-associated plasmin activity that peaked between 8 and 24 hours and correlated with an increase in apoptosis (Fig 3C).

We also tested whether TM5441 at concentrations of 1 μM to 25 μM would affect the sensitivity of HT1080 and HCT116 cells to several chemotherapeutic agents (doxorubicin, etoposide, oxaloplatin, and 5-fluorouracil (5-FU). The data revealed that TM5441 did not potentiate the activity of these chemotherapeutic agents at 1 μM. Even when used at a higher concentration (25 μM), TM5441 did not potentiate the cytotoxic activity of doxorubicin (S2 Fig).

### TM5275 and TM5441 induced mitochondrial depolarization

**Fig 4:**
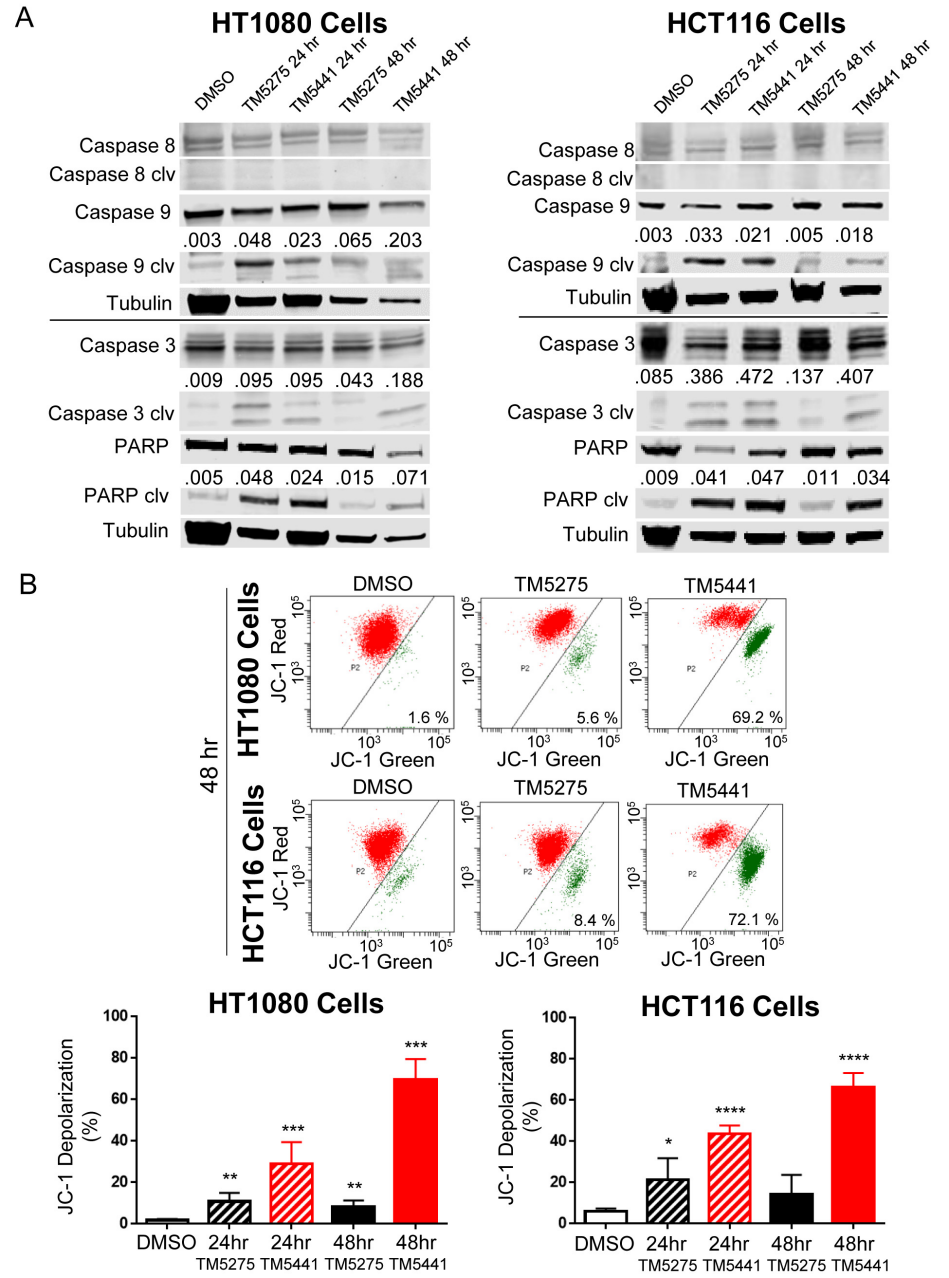
Treatment with TM5275 or TM5441 increases intrinsic apoptosis. **A**. Representative immunoblots for HT1080 and HCT116 cells treated with 25 μM or 50 μM TM5275 or TM5441 as indicated for caspase 8, 9, 3, PARP, and their cleavage products, n = 3. The numbers over the cleaved protein bands indicate the ratio of the cleaved protein pixel density to the corresponding tubulin control band pixel density. **B**. Representative JC-1 FACS plots of cells treated for 48 hours with 50 μM TM5275 or TM5441. The graphs show the average % (± SD) of JC-1 membrane depolarization in control DMSO-treated and TM inhibitor-treated cells n = 3. * indicates *P* values compared to DMSO controls < 0.05, ** indicates *P* < 0.01, *** indicates *P* < 0.001, and **** indicates *P* < 0.0001.

To explore the relative contribution of the extrinsic and intrinsic apoptotic pathways in TM inhibitor-induced apoptosis, we examined the effect of these inhibitors on the cleavage (activation) of caspase 3, 8 and 9 by Western blot. The data (Fig 4A) revealed an absence of caspase 8 activation in both cell lines upon treatment with TM5275 or TM5441. In contrast they indicated activation of caspase 9 and 3 and a corresponding cleavage of Poly adipo-ribose polymerase (PARP), a substrate for caspase 3, which was consistent with an activation of the intrinsic apoptotic pathway by TM inhibitors. This was confirmed by the examination of mitochondrial membrane depolarization in HT1080 and HCT116 cells treated with TM inhibitors (50 μM) (Fig 4B). The data indicated an increase in mitochondrial depolarization in cells treated with TM5275 and TM5441 with a much more pronounced effect of TM5441 as previously observed in caspase 3/7 activity assays (Fig 3A). These results indicated that TM5275, and in particular TM5441, are potent stimulators of intrinsic apoptosis in tumor cells.

### TM5441 has a biological effect *in vivo*

**Fig 5:**
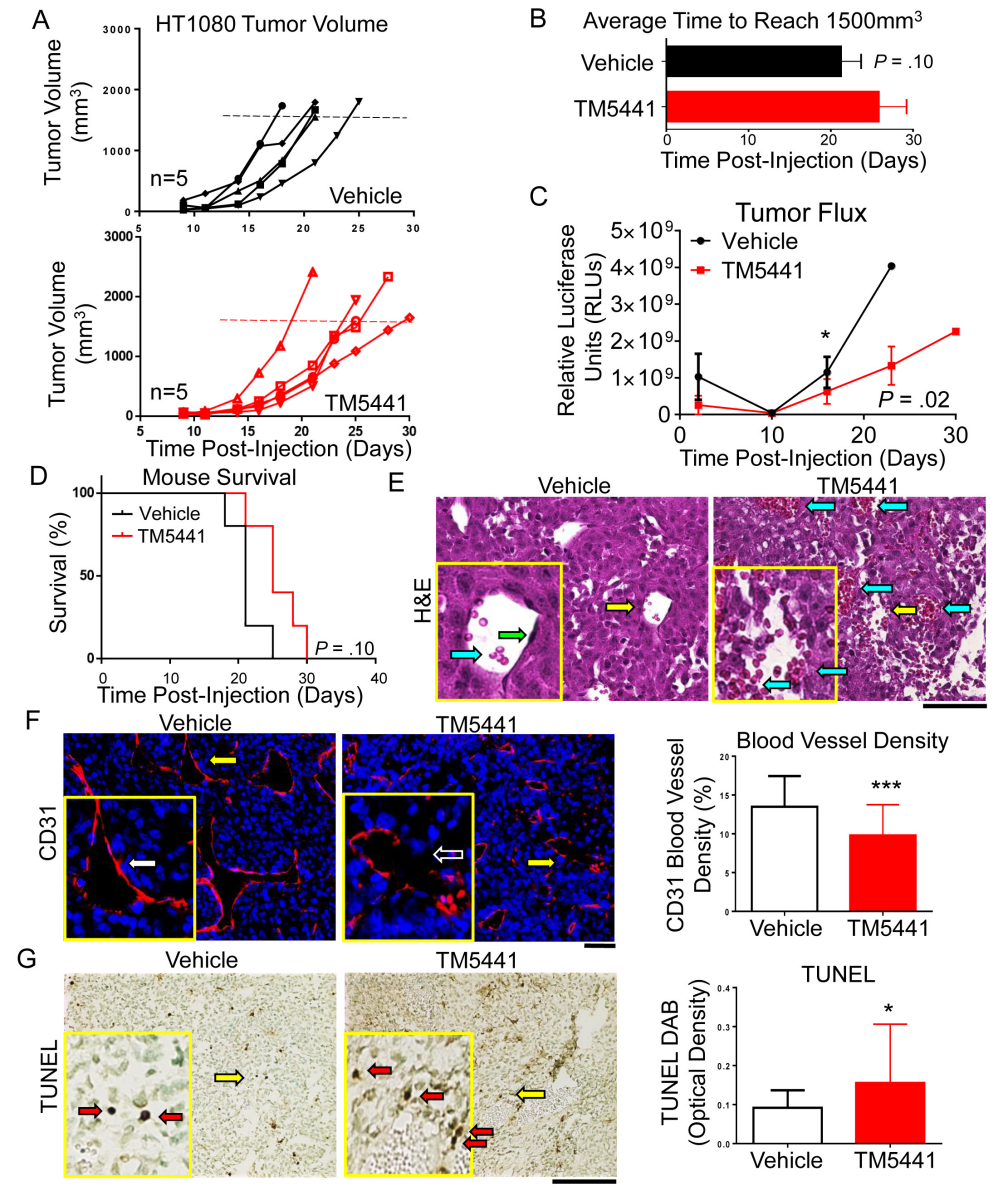
Pre-clinical activity of TM5441 *in vivo*. **A**. Individual tumor volume was plotted over time (days) for vehicle and TM5441 (20 mg/kg/day) treated mice (n = 5 per group). **B**. Average time (±SD) to reach maximal tumor growth for vehicle and TM5441-treated mice was plotted over time (days). **C**. Average tumor relative luciferase units (RLUs) (±SD) for mice treated with vehicle or TM5441 was plotted over time (days). **D**. Mouse survival (%) was plotted for mice treated with vehicle (black line) or TM5441 (red line) over time (days). **E**. Representative histology sections of tumors stained with H&E. Yellow arrows indicate the area selected for the inset panels. Blue arrows indicate hemorrhagic areas with red blood cells. Green arrows indicate EC lining the vessels. Scale bar = 50 μm. **F**. Representative histological sections of tumors stained for CD31 (red) as described in Materials and Methods and counterstained with nuclear 4’6’-diamidino-2-phenylindole (DAPI) (blue) staining. Yellow arrows indicate the area selected for the inset panels. White arrows indicate CD31 positive cells lining the vessel wall. Clear arrows with a white outline indicate the absence of distinct CD31 positive cells. The graph shows the percentage of CD31 positive staining (± SD, n = 5 tumors per group with 5 sections per tumor, n = 25 total per group). Scale bar = 50 μm. **G**. Representative histology sections of tumors stained with TUNEL as described in Materials and Methods. TUNEL staining (brown) counterstained with methyl green (green). Yellow arrows indicate the area selected for the inset panels. Red arrows indicate TUNEL positive cells. The graph shows the average TUNEL optical density (± SD, n = 5 tumors per group with 5 sections per tumor, n = 25 total per group). Scale bar = 50 μm. * indicates *P* values compared to vehicle control < 0.05, and *** indicates *P* < 0.001.

Due to its higher apoptotic effect *in vitro*, TM5441 was selected to test its anti-tumor activity *in vivo* in mice xenotransplanted with HT1080 cells (Fig 5). This experiment revealed that tumor-bearing mice treated with TM5441 (20 mg/kg daily) showed a trend to develop slower growing tumors that reached a volume of 1,500 mm^3^ at an average of 25.8 (± 3.4) days vs. 21.2 (± 2.5) days for the control group (*P* value = 0.10) (Fig 5A and 5B). However, there was a statistically significant decrease in bioluminescence activity (relative luciferase units (RLUs) as an indicator of tumor cell viability) over time in the TM5441-treated group (Fig 5C). The effect of TM5441 treatment on survival showed a trend toward an increase in survival in the TM5441-treated group that was, however, not statistically significant (*P* value = 0.10) (Fig 5D). A histological analysis of these tumors by hematoxylin and eosin (H&E) indicated the presence of larger hemorrhagic areas with disrupted vasculature in tumors from TM5441-treated mice. This disruption of the tumor vasculature was confirmed by staining for CD31 (PECAM) which revealed multiple areas of discontinuity in EC comprising blood vessels and a statistically significant decrease in blood vessel density in the TM5441-treated group (*P* value = 0.002) (Fig 5F). There was also an increased amount of TUNEL positive staining (a marker of tumor cell apoptosis) in tumors from TM5441-treated mice (*P* value = 0.05) (Fig 5G). Taken together, the data demonstrate that TM5441 has a biological activity on the tumor vasculature and on tumor cells *in vivo* that, however, was not sufficient to significantly affect tumor growth. Similar results were seen in our *in vivo* HCT116 tumor model (S3 Fig). Pharmacokinetics studies revealed in mice treated with 20 mg/kg an average peak concentration of 11.4 μM (only slightly lower than the IC_50_ of 13.9 μM for HT1080) and undetectable trough levels (S4 Fig). Administration of 50 mg/kg and 100 mg/kg of TM5441, although increasing the peak plasma concentration levels to 15.0 μM and 35.6 μM, respectively, did not significantly increase the trough levels and did not affect tumor growth (S5 Fig). Interestingly, the administration of TM5441 had no systemic effect on bleeding time in mice treated with 50 or 100 mg/kg (S6A Fig). In an *in vitro* human plasma clot lysis assay TM5275 effectively inhibited PAI-1 from maintaining the clot and allowed lysis to occur similarly to the addition of tPA (S6B Fig).

### Inhibited HUVEC branching with TM5275 and TM5441

**Fig 6:**
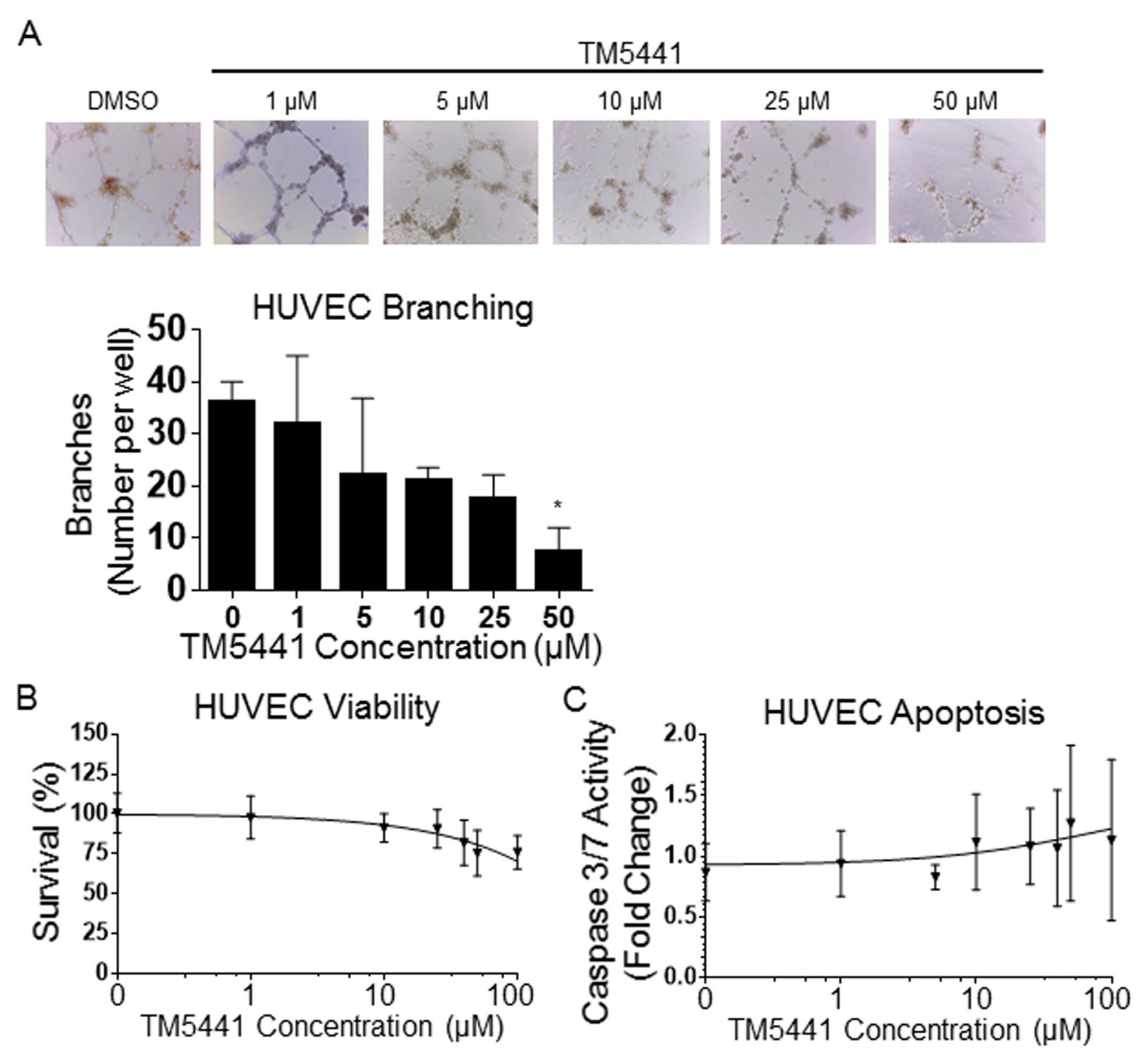
TM5441 inhibits EC branching morphogenesis. **A**. Representative photographs of HUVEC cultured in Matrigel with the indicated concentrations of TM5441. The graph represents the average number of HUVEC branch points (± SD) at the indicated TM5441 concentrations, n = 3. **B**. The graph represents the average % survival (± SD) in HUVEC at the indicated concentrations of TM5441, n = 3. **C**. The graph represents the average fold change of caspase 3/7 activity (± SD) compared to DMSO controls for the indicated concentrations of TM5441, n = 3. * indicates *P* values compared to DMSO control wells < 0.05.

The disruptive effect of TM5441 on the tumor vasculature *in vivo* was further investigated on EC *in vitro* (Fig 6). This analysis revealed a dose-dependent inhibition of TM5441 on branching morphogenesis of HUVEC plated in 3D Matrigel cultures (Fig 6A). This effect, however, was not related to a direct effect on EC viability as TM5441 had no effect on HUVEC survival (Fig 6B) and apoptosis (Fig 6C) at a concentration of 50 μM, whereas, it had a significant effect on branching morphogenesis *in vitro*. A similar effect on EC was observed with TM5275 (S1 and S7 Figs). The data thus indicate that TM inhibitors have a significant vascular disruption activity that is independent of their apoptotic activity.

## Discussion

This data represents a first *in vivo* analysis of TM5441 PAI-1 inhibitor activity in cancer. TM5275 and TM5441 induced intrinsic apoptosis in several human cancer cell lines and inhibited EC branching in a manner that was independent from their apoptotic activity on EC *in vitro*. These *in vivo* results in HT1080 and HCT116 xenograft models showed that although TM5441 had a vascular disruptive effect (and a trend of decreased tumor growth and increased survival in the HT1080 model), these effects were not sufficient to affect tumor growth even as we documented a significant decrease in TUNEL staining *in vivo*.

As a basis for comparison, the IC_50_ of TM5275 and TM5441 treatment is similar to the IC_50_ of PAI-039, another previously reported PAI-1 inhibitor. The IC_50_ measured by tPA-dependent hydrolysis for the compounds were 8.37 μM for PAI-749, which is a more potent derivative of PAI-039, and 6.95 μM for TM5275 [15]. When defined on the basis of cell viability, the IC_50_ of PAI-039 was calculated from previous data to be 29 μM and 32 μM for HT1080 and HCT116 cells, respectively [10], which is in the range of the IC_50_ found for TM5275 and TM5441. There was no correlation between the IC_50_ of the TM compounds and the total PAI-1 levels measured in the cell lysates. This suggested that other factors besides PAI-1 played a role, which may include membrane-associated plasmin, uPA, or sensitivity to apoptosis that all contributed to the control of cell viability.

The effect of TM5275 and TM5441 on intrinsic apoptosis was somewhat unanticipated in view of our previous work that demonstrated a protective effect of PAI-1 on Fas-L-mediated extrinsic apoptosis [10], suggesting that the effect of these inhibitors may not involve control over Fas-L-mediated apoptosis. A similar observation with TM5275 has been reported *in vitro* in ovarian cancer cells that demonstrated TM5275 induced apoptosis through activation of the intrinsic apoptotic pathway [19]. Studies have shown that PAI-1 is able to directly bind to caspase 3, thus influencing activation of apoptosis [25–31]. Although the exact mechanism is unknown it has been suggested that PAI-1 in complex with uPA/uPAR is internalized and upon its intracellular release inhibits caspase 3 [26]. Indirect evidence for such intracellular inhibitory activity of PAI-1 was also noted in a model of vascular smooth muscle cells from ApoE^-/-^PAI-1^-/-^ mice showing higher apoptosis associated with increased plasmin and active caspase-3 *in vivo* [30]. Another study found that proliferating PAI-1^-/-^ endothelial cells have increased Akt activation and decreased levels of procaspase 3 and caspase 3 leading to increased survival [28]. These studies demonstrate a role for intracellular PAI-1 influencing the balance of proliferation and apoptosis [31] that may be affected by TM inhibitors.

Our studies bring important insight into the limitations and challenges of targeting PAI-I in cancer [32]. A first limitation is the high concentrations (μM) of TM inhibitors needed to reach a biological effect *in vitro* and *in vivo*. The average experimental one-hour peak plasma concentration for TM5441 was near the IC_50_ for HT1080 cells and about half the IC_50_ for HCT116 cells. This suggests that the lack of a significant effect on tumor growth *in vivo* is related to the inability to reach sustainable inhibitor plasma concentrations within the active range. In contrast to thrombotic diseases where blocking PAI-1 needs to be rapidly but also transiently achieved, targeting PAI-1 in cancer requires the chronic administration of an inhibitor and thus a much more favorable pharmacokinetic profile where effective concentrations can be achieved for longer periods of time. This will require the development of small molecule inhibitors with activity in the nM range and with a much longer half-life.

The effect of TM5441 on the tumor vasculature deserves further investigation. It is of particular interest that vascular disruption was observed *in vitro* and *in vivo* at concentrations where TM5441 had no significant apoptotic effect on HUVEC survival and apoptosis *in vitro*. This suggested that future PAI-1 inhibitors could potentially modulate tumor angiogenesis by inhibiting the formation of new vessels without disrupting established blood vessels in normal tissues limiting systemic toxicity. A potential explanation is that inhibition of PAI-1 at these concentrations is sufficient to affect EC motility and/or EC tight junctions but not to induce apoptosis which may require higher levels of plasmin activity. A similar effect was seen with another PAI-1 inhibitor SK-216 that was shown not to affect HUVEC proliferation, but to inhibit migration and tube formation *in vitro* [33]. It is unknown how vascular disruption may alter the effectiveness of the inhibitor on tumor cell viability and apoptosis. This effect on vascular disruption is being further investigated in our laboratory.

A major concern with the use of PAI-1 inhibition in cancer has been its potential toxic effect in promoting fibrinolysis and inducing severe bleeding upon chronic administration [34, 35]. Interestingly, our studies demonstrated that even administration of TM5441 to mice at 100 mg/kg/day did not increase fibrinolysis to a point where it impaired blood clot formation *in vivo* as indicated by an absence of effect on bleeding time [36]. This is also consistent with the fact that humans with a PAI-1 deficiency are rarely identified as they do not have an increase in spontaneous bleeding. It is only under stressed conditions such as surgery or repeated miscarriages in women, that they express a decrease in blot clot formation [37]. This suggests that chronic administration of PAI-1 may be tolerable.

In summary, our analysis of the pre-clinical efficacy of TM inhibitors of PAI-1 brings important and novel information on their activity in cancer, but also tolerability that should be helpful in the future design of more effective inhibitors.

## Supporting Information

S1 Fig Cell viability in cells treated with TM5275. The indicated human cell lines were treated with increasing concentrations of TM5275 (black). The top row of cells (HUVEC, bone marrow-derived stem cells (BMSC), and foreskin fibroblasts) are benign and all other cell lines are malignant. Viability was measured by relative luminescence units for increasing concentrations of TM5275 and were graphed as % survival (± SD) to DMSO-treated cells. Data was plotted and a best fit line was drawn, n = 1, except HUVEC n = 3.
(TIF)

S2 Fig Cell viability in cancer cells treated with TM5441 and chemotherapy. **A.** The indicated cell lines were treated with increasing concentrations of the indicated chemotherapy (black) alone or in combination with 1 μM TM5441 (red). Viability was measured by relative luminescence units for increasing concentrations of chemotherapy and graphed as % survival (± SD) to control-treated cells. Data was plotted and a best fit line was drawn, n = 1, except doxorubicin n = 3. **B.** The cell lines were treated with increasing concentrations of TM5441 (black) alone or in combination with 0.05 μM doxorubicin (blue) or 0.1 μM doxorubicin (red). Viability was measured by relative luminescence units for increasing concentrations of the drug and graphed as % survival (± SD) to control-treated cells. Data was plotted and a best fit line was drawn, n = 2.
(TIF)

S3 Fig Pre-clinical activity of TM5441 in HCT116 cells *in vivo*. **A**. Individual tumor volume was plotted over time (days) for vehicle and TM5441 (20 mg/kg/day) treated mice (n = 5 per group). **B**. Average time (±SD) to reach maximal tumor growth for vehicle and TM5441-treated mice was plotted over time (days). **C**. Mouse survival (%) was plotted for mice treated with vehicle (black line) or TM5441 (red line) over time (days). **D**. Representative histology sections of tumors stained with H&E. Scale bar = 50 μm. **E**. Representative histological sections of tumors stained for CD31 (red) as described in Materials and Methods and counterstained with nuclear DAPI (blue) staining. The graph shows the percentage of CD31 positive staining (± SD, n = 5 tumors per group with 5 sections per tumor, n = 25 total per group). Scale bar = 50 μm. ** indicates *P* values compared to vehicle control < 0.01.
(TIF)

S4 Fig Plasma concentrations of TM5441 in treated mice. The average peak (one hour after administration) and trough (one hour before next administration) plasma concentrations from mice treated at the indicated doses were plotted (± SD, n = 5–10 mice per group).
(TIF)

S5 Fig Pre-clinical activity of TM5441 *in vivo*. **A**. Individual tumor volume was plotted over time (days) for vehicle and TM5441 (50 mg/kg/day) treated mice (n = 10 per group). **B**. Individual tumor volume was plotted over time (days) for vehicle and TM5441 (100 mg/kg/day) treated mice (n = 10 per group).
(TIF)

S6 Fig Murine bleeding time and human plasma clot lysis. **A.** Bleeding time (minutes) values in mice treated with vehicle or TM5441 at the indicated doses. The bleeding was performed one hour (peak time) after the last administration of TM5441. The average bleeding time for each group is indicated with a solid black horizontal line. (50 mg/kg daily *P* value = 0.48, n = 10) (100 mg/kg daily *P* value = 0.24, n = 5). **B.** Comparative analysis of human plasma clot lysis effect between the indicated compounds. Human plasma clot lysis assay medium included 50% pooled human normal plasma, 2.4 nM human active PAI-1, 2 nM human 2-chain t-PA, 0.1 M NaCl, 20mM Tris-HCl pH 7.5, 10 mM CaCl_2_ and 2.5 U/mL human thrombin at final concentrations. PAI-1 inhibitors were dissolved in DMSO (final 0.1%) and anti-human PAI-1 in phosphate-buffered saline (PBS). The reaction was initiated by the addition of CaCl_2_ and thrombin and then monitored at OD 405 nm with a microplate reader at room temperature.
(TIF)

S7 Fig TM5275 inhibits endothelial cell branching morphogenesis. **A**. Representative photographs of HUVEC cultured in Matrigel under the indicated concentrations of TM5275. The graph represents the average number of HUVEC branch points (± SD) at the indicated TM5275 concentrations, n = 3. **B**. The graph represents the mean fold change of caspase 3/7 activity (\ SD) in HUVEC at the indicated concentrations of TM5275 compared to DMSO controls, n = 3. * indicates *P* values compared to DMSO controls < 0.05 and ** indicates *P* < 0.01.
(TIF)

S1 Materials and Methods Cell culture. Human foreskin fibroblast cells were provided by Dr. Tai-Lan Tuan (Children’s Hospital Los Angeles, Los Angeles, CA). Bone marrow-derived mesenchymal stem cells (BMSC), prostate adenocarcinoma (PC-3), rhabdomyosarcoma (A204), ovarian adenocarcinoma (SK-OV-3), liver hepatocellular carcinoma (Hep G2), lung carcinoma (A549), osteosarcoma (SaOs2), glioblastoma (U87), melanoma primary tumor (M24), and metastatic derivative of M24 (M24met) human cancer cell lines were purchased from ATCC. The aforementioned cells were grown in DMEM medium supplemented with 10% fetal bovine serum.
(DOCX)

S1 Table IC_50_ values for TM5275 and TM5441 treated cell lines. Mean IC_50_ values for TM5275 and TM5441 determined from the analysis shown in Fig 1, (n = 3). The total cell-associated PAI-1 level for each cell line is also listed.
(PDF)
