# Supplementary figures and images for: Small Molecule Inhibitors of Plasminogen Activator Inhibitor-1 Elicit Anti-Tumorigenic and Anti-Angiogenic Activity
Source: PLoS One. 2015 Jul 24;10(7):e0133786. doi: 10.1371/journal.pone.0133786 (PMC4514594; doi:10.1371/journal.pone.0133786)

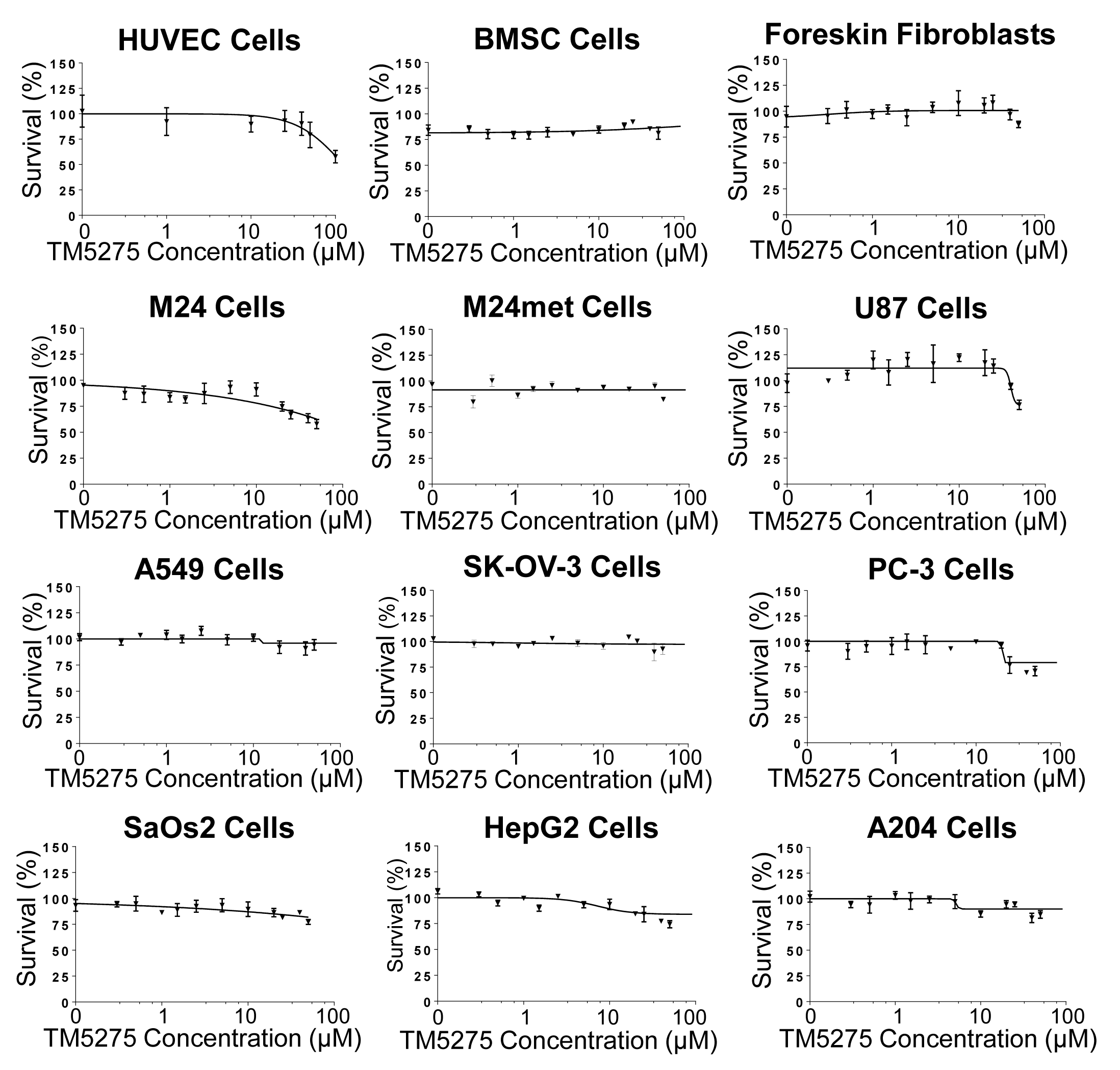

Supplement: S1 Fig — The indicated human cell lines were treated with increasing concentrations of TM5275 (black). The top row of cells (HUVEC, bone marrow-derived stem cells (BMSC), and foreskin fibroblasts) are benign and all other cell lines are malignant. Viability was measured by relative luminescence units for increasing concentrations of TM5275 and were graphed as % survival (± SD) to DMSO-treated cells. Data was plotted and a best fit line was drawn, n = 1, except HUVEC n = 3. (TIF) [file pone.0133786.s001.tif]

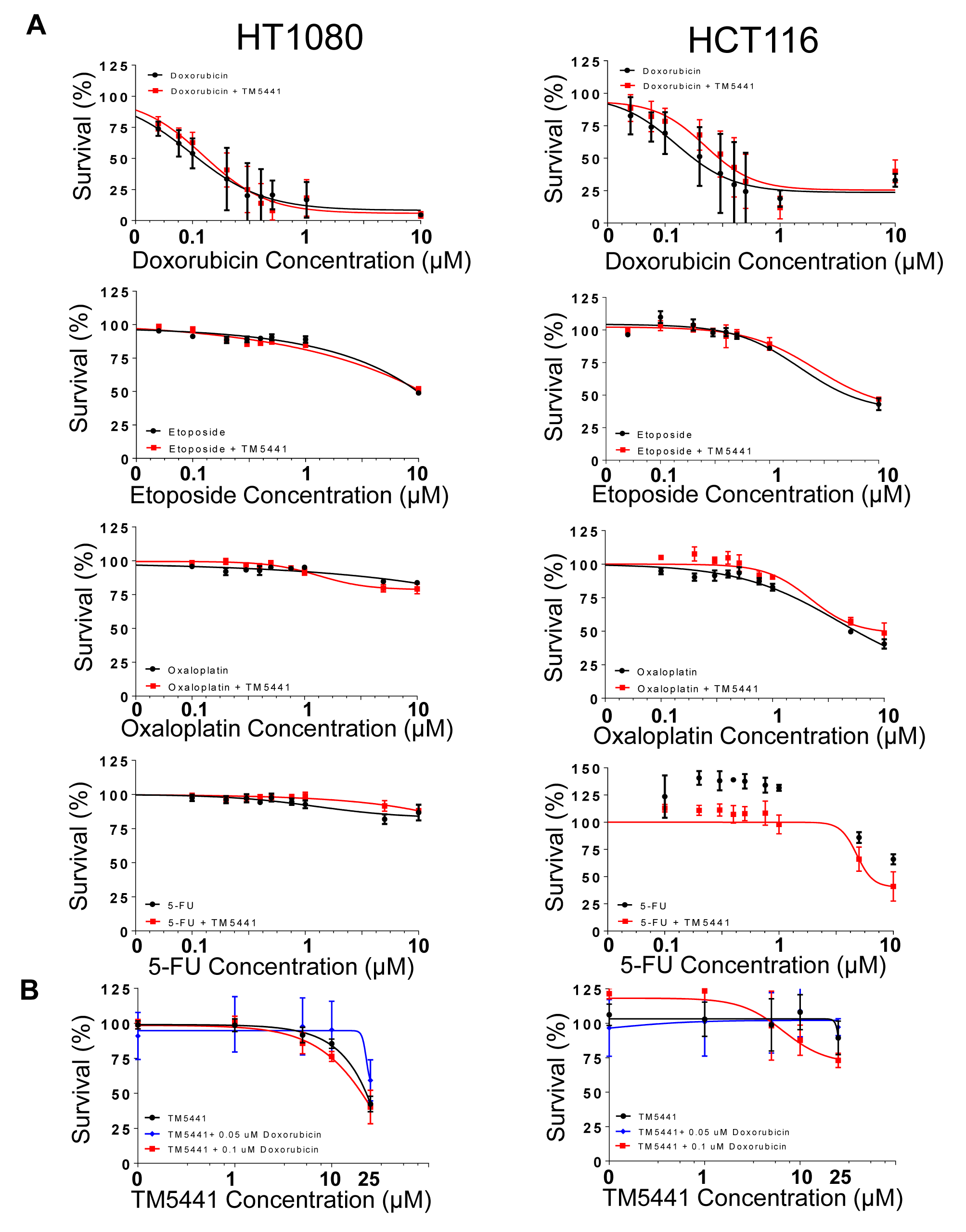

Supplement: S2 Fig — A. The indicated cell lines were treated with increasing concentrations of the indicated chemotherapy (black) alone or in combination with 1 μM TM5441 (red). Viability was measured by relative luminescence units for increasing concentrations of chemotherapy and graphed as % survival (± SD) to control-treated cells. Data was plotted and a best fit line was drawn, n = 1, except doxorubicin n = 3. B. The cell lines were treated with increasing concentrations of TM5441 (black) alone or in combination with 0.05 μM doxorubicin (blue) or 0.1 μM doxorubicin (red). Viability was measured by relative luminescence units for increasing concentrations of the drug and graphed as % survival (± SD) to control-treated cells. Data was plotted and a best fit line was drawn, n = 2. (TIF) [file pone.0133786.s002.tif]

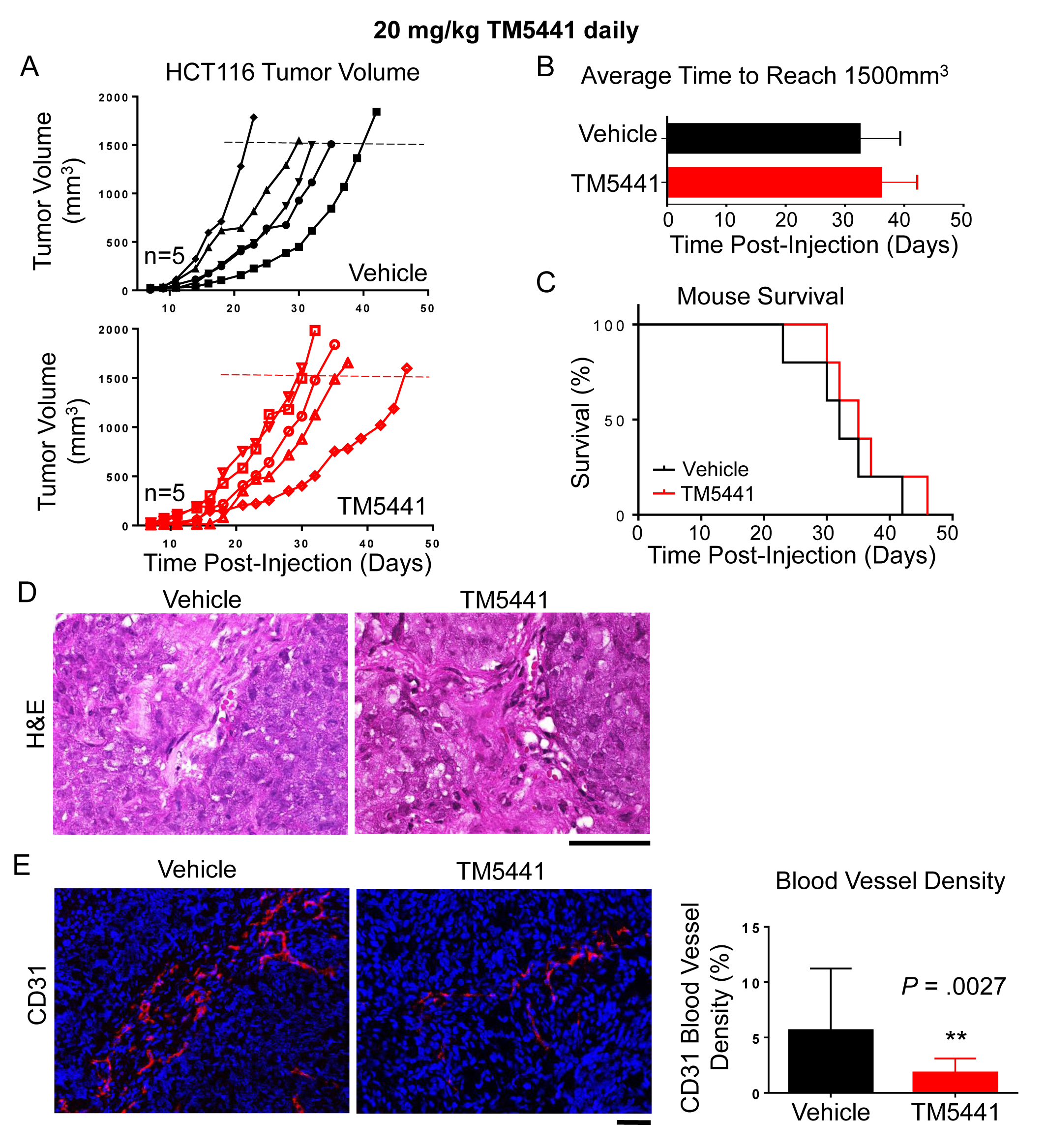

Supplement: S3 Fig — A. Individual tumor volume was plotted over time (days) for vehicle and TM5441 (20 mg/kg/day) treated mice (n = 5 per group). B. Average time (±SD) to reach maximal tumor growth for vehicle and TM5441-treated mice was plotted over time (days). C. Mouse survival (%) was plotted for mice treated with vehicle (black line) or TM5441 (red line) over time (days). D. Representative histology sections of tumors stained with H&E. Scale bar = 50 μm. E. Representative histological sections of tumors stained for CD31 (red) as described in Materials and Methods and counterstained with nuclear DAPI (blue) staining. The graph shows the percentage of CD31 positive staining (± SD, n = 5 tumors per group with 5 sections per tumor, n = 25 total per group). Scale bar = 50 μm. ** indicates P values compared to vehicle control < 0.01. (TIF) [file pone.0133786.s003.tif]

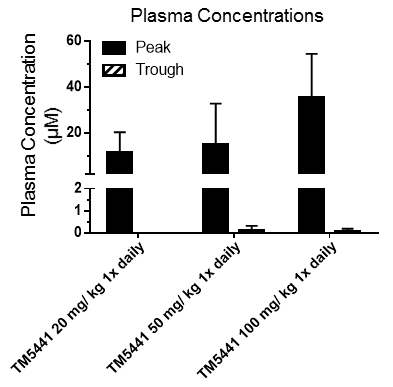

Supplement: S4 Fig — The average peak (one hour after administration) and trough (one hour before next administration) plasma concentrations from mice treated at the indicated doses were plotted (± SD, n = 5–10 mice per group). (TIF) [file pone.0133786.s004.tif]

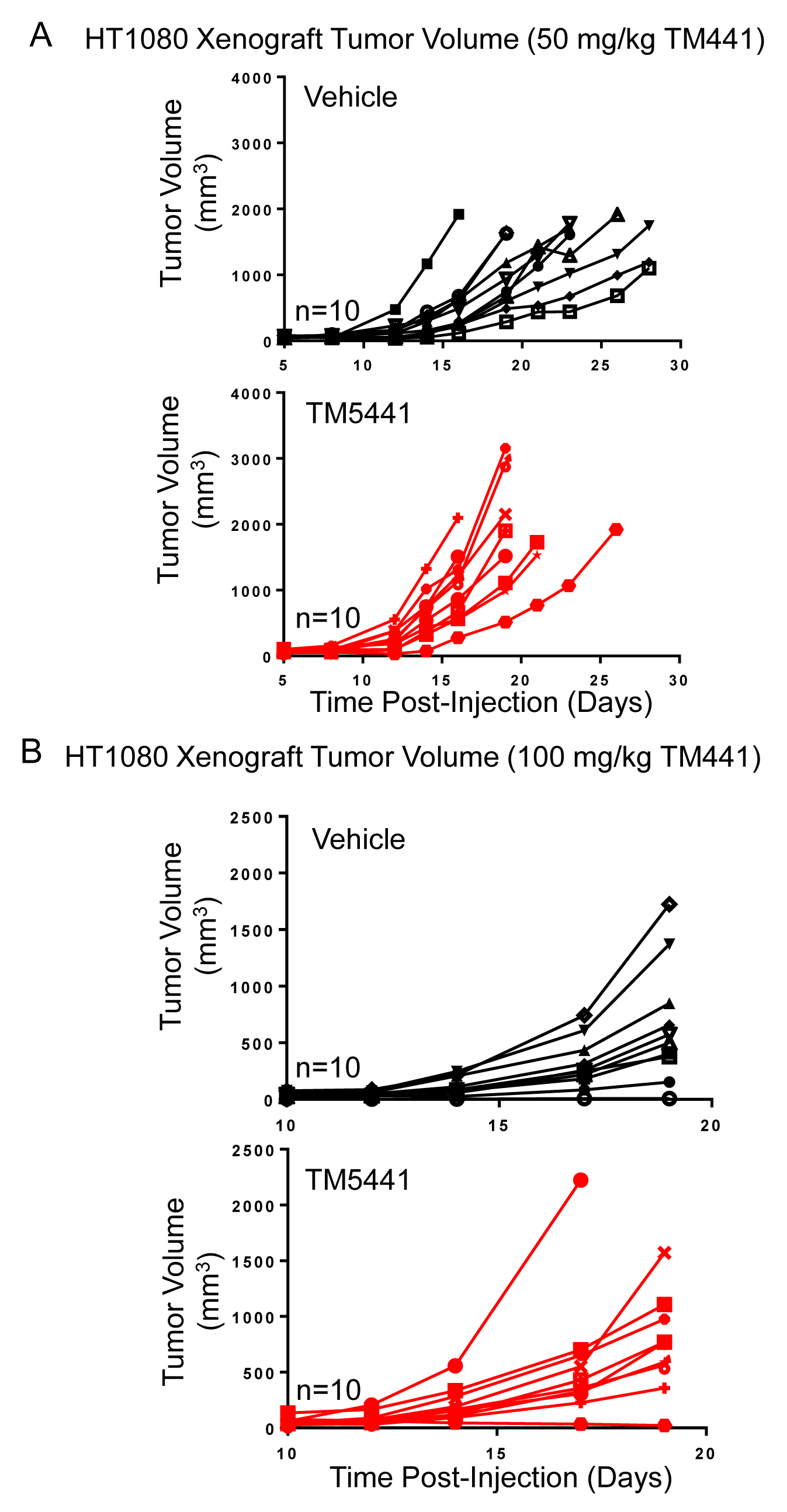

Supplement: S5 Fig — A. Individual tumor volume was plotted over time (days) for vehicle and TM5441 (50 mg/kg/day) treated mice (n = 10 per group). B. Individual tumor volume was plotted over time (days) for vehicle and TM5441 (100 mg/kg/day) treated mice (n = 10 per group). (TIF) [file pone.0133786.s005.tif]

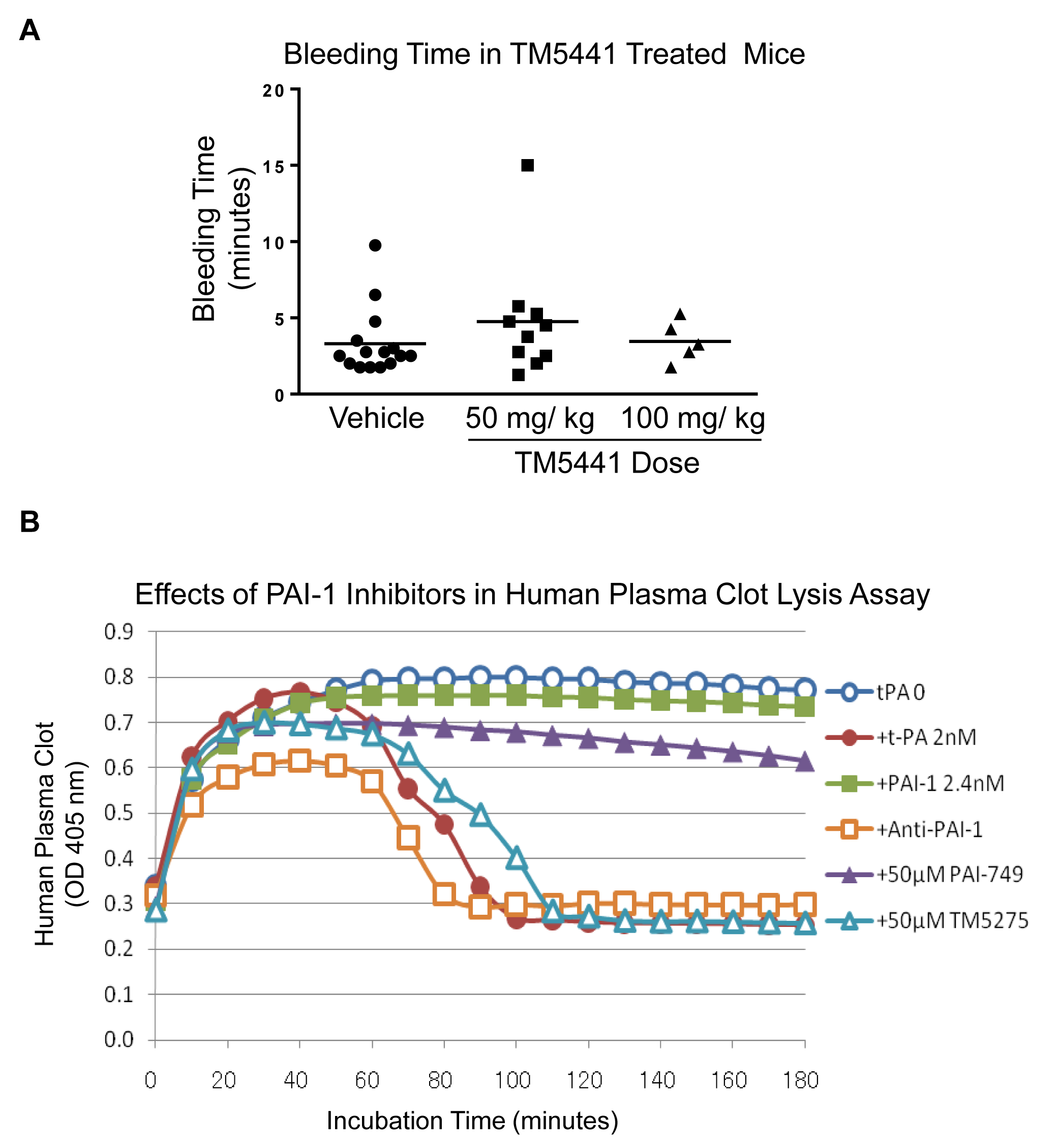

Supplement: S6 Fig — A. Bleeding time (minutes) values in mice treated with vehicle or TM5441 at the indicated doses. The bleeding was performed one hour (peak time) after the last administration of TM5441. The average bleeding time for each group is indicated with a solid black horizontal line. (50 mg/kg daily P value = 0.48, n = 10) (100 mg/kg daily P value = 0.24, n = 5). B. Comparative analysis of human plasma clot lysis effect between the indicated compounds. Human plasma clot lysis assay medium included 50% pooled human normal plasma, 2.4 nM human active PAI-1, 2 nM human 2-chain t-PA, 0.1 M NaCl, 20mM Tris-HCl pH 7.5, 10 mM CaCl2 and 2.5 U/mL human thrombin at final concentrations. PAI-1 inhibitors were dissolved in DMSO (final 0.1%) and anti-human PAI-1 in phosphate-buffered saline (PBS). The reaction was initiated by the addition of CaCl2 and thrombin and then monitored at OD 405 nm with a microplate reader at room temperature. (TIF) [file pone.0133786.s006.tif]

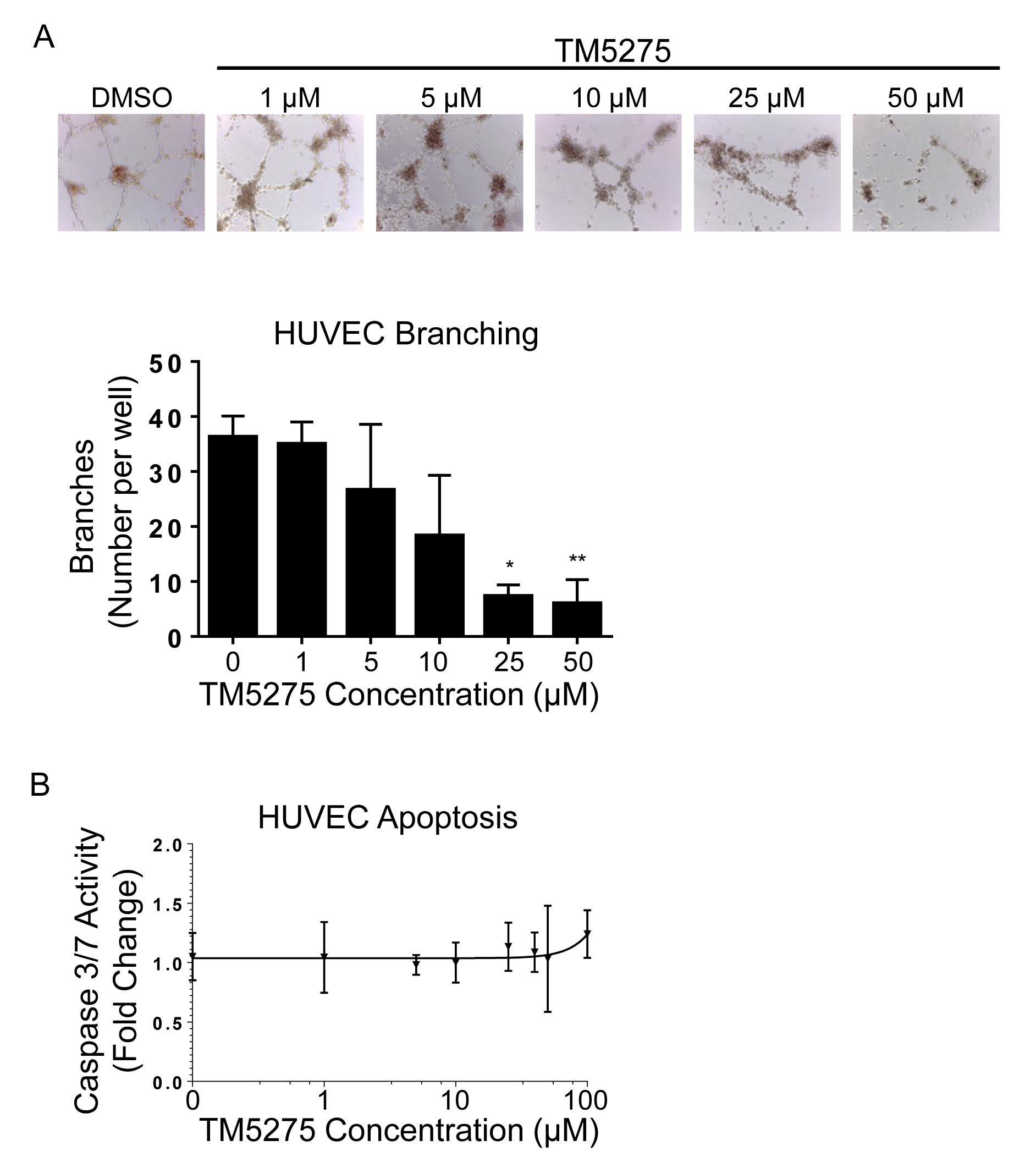

Supplement: S7 Fig — A. Representative photographs of HUVEC cultured in Matrigel under the indicated concentrations of TM5275. The graph represents the average number of HUVEC branch points (± SD) at the indicated TM5275 concentrations, n = 3. B. The graph represents the mean fold change of caspase 3/7 activity (\ SD) in HUVEC at the indicated concentrations of TM5275 compared to DMSO controls, n = 3. * indicates P values compared to DMSO controls < 0.05 and ** indicates P < 0.01. (TIF) [file pone.0133786.s007.tif]
