# Supplementary material for: Small Molecule Inhibitors of Plasminogen Activator Inhibitor-1 Elicit Anti-Tumorigenic and Anti-Angiogenic Activity
Source: PLoS One. 2015 Jul 24;10(7):e0133786. doi: 10.1371/journal.pone.0133786 (PMC4514594; doi:10.1371/journal.pone.0133786)
Supplement: S1 Materials and Methods — Human foreskin fibroblast cells were provided by Dr. Tai-Lan Tuan (Children’s Hospital Los Angeles, Los Angeles, CA). Bone marrow-derived mesenchymal stem cells (BMSC), prostate adenocarcinoma (PC-3), rhabdomyosarcoma (A204), ovarian adenocarcinoma (SK-OV-3), liver hepatocellular carcinoma (Hep G2), lung carcinoma (A549), osteosarcoma (SaOs2), glioblastoma (U87), melanoma primary tumor (M24), and metastatic derivative of M24 (M24met) human cancer cell lines were purchased from ATCC. The aforementioned cells were grown in DMEM medium supplemented with 10% fetal bovine serum. (DOCX) [file pone.0133786.s008.docx]

**Supporting Information Materials and Methods**

**Cell culture**

Human foreskin fibroblast cells were provided by Dr. Tai-Lan Tuan (Children’s Hospital Los Angeles, Los Angeles, CA). Bone marrow-derived mesenchymal stem cells (BMSC), prostate adenocarcinoma (PC-3), rhabdomyosarcoma (A204), ovarian adenocarcinoma (SK-OV-3), liver hepatocellular carcinoma (Hep G2), lung carcinoma (A549), osteosarcoma (SaOs2), glioblastoma (U87), melanoma primary tumor (M24), and metastatic derivative of M24 (M24met) human cancer cell lines were purchased from ATCC. The aforementioned cells were grown in DMEM medium supplemented with 10% fetal bovine serum.
