# Supplementary material for: Small Molecule Inhibitors of Plasminogen Activator Inhibitor-1 Elicit Anti-Tumorigenic and Anti-Angiogenic Activity
Source: PLoS One. 2015 Jul 24;10(7):e0133786. doi: 10.1371/journal.pone.0133786 (PMC4514594; doi:10.1371/journal.pone.0133786)
Supplement: S1 Table — Mean IC50 values for TM5275 and TM5441 determined from the analysis shown in Fig 1, (n = 3). The total cell-associated PAI-1 level for each cell line is also listed. (PDF) [file pone.0133786.s009.pdf]

Cell Line	Cell Type	TM5275 IC_{50} (μM)	TM5441 IC_{50} (μM)	PAI-1 level (ng/ mg)
HT1080	Fibrosarcoma	21.7	13.9	692
HCT 116	Colorectal Carcinoma	37.4	27.3	26.9
Daoy	Desmoplastic Cerebellar Medulloblastoma	9.70	29.1	494
MDA-MB-231	Breast Adenocarcinoma	60.3	47.0	729
Jurkat	T Lymphocytes from Acute Lymphocytic Leukemia	25.3	51.1	19.9
